# Invasive mechanical ventilation in COVID-19 patient management: the experience with 469 patients in Wuhan

**DOI:** 10.1186/s13054-020-03044-9

**Published:** 2020-06-16

**Authors:** Jing Hua, Chenchen Qian, Zhibing Luo, Qiang Li, Feilong Wang

**Affiliations:** 1 Department of Pulmonary and Critical Care Medicine, Shanghai East Hospital, Tongji University School of Medicine, Shanghai, China; 2 Department of Internal Medicine, UPMC Pinnacle Hospital, Harrisburg, PA USA

Dear Editor,

Since the first case of COVID-19 was reported in Wuhan, this new respiratory disease has evolved rapidly and been found in almost all the countries in the world. From our clinical experiences during managing COVID-19 patients, we observed an extremely high fatality rate in invasive ventilation (IV) patients which was astonishing and unexpected.

To validate our assumption, we collected and analyzed the data of 469 ICU COVID-19 patients who were hospitalized from February 2020 to the end of March in 13 ICUs in Wuhan. At the time of data collection, all of the patients were either discharged or deceased (Table 1).

**Table 1:** Centers and study periods

Centers	Study period	No. of cases
Huoshenshan Hospital (2 ICUs)	February 2 to March 31	118
Leishenshan Hospital (2 ICUs)	February 23 to March 31	41
Guanggu Hospital (2 ICUs)	February 21 to March 25	40
Taikang Hospital (2 ICUs)	February 11 to March 21	42
Zhongfaxincheng Hospital (3 ICUs)	February 8 to March 15	147
Wuhan Fifth Hospital (1 ICU)	February 20 to March 31	21
Union Hospital (1 ICU)	February 15 to March 31	60

Clinical features, laboratory results on admission, and outcomes are shown in Table 2. We found that the mortality rate in the IV group was 92%, compared to the other two groups (6.4% in the NV group, 40.8% in the NIV group). Furthermore, patients in the IV group developed a higher rate of severe comorbidities such as acute kidney injury (AKI) which required continuous renal replacement therapy (CRRT) (26.5%) compared to the NV (2.9%) and NIV (5.3%) groups. Moreover, 10 patients (8.8%) in the IV group received ECMO implementation.

**Table 2:** Clinical features, laboratory results on admission, and outcomes of the study patients

	All (*n* = 469)	No ventilation (*n* = 204)	Invasive ventilation (*n* = 113)	Noninvasive ventilation (*n* = 152)	*P*
Age	68 ± 13	67 ± 15	71 ± 10	67 ± 13	0.030
Sex					0.034
Male	266 (56.7)	108 (52.9)	76 (67.3)	82 (53.9)	
Female	203 (43.3)	96 (47.1)	37 (32.7)	70 (46.1)	
Comorbidities, no. (%)					
Hypertension	240 (51.4)	99 (48.5)	56 (49.6)	85 (56.7)	0.288
Diabetes	110 (23.6)	41 (20.1)	28 (24.8)	41 (27.3)	0.268
Coronary artery disease	84 (18.0)	44 (21.6)	20 (17.9)	20 (13.3)	0.137
Chronic obstructive lung disease	52 (11.1)	13 (6.4)	8 (7.1)	31 (20.7)	< 0.001
Chronic kidney disease	42 (9.0)	21 (10.3)	8 (7.1)	13 (8.7)	0.623
Laboratory results on admission					
White blood cell count, × 10^9^/L	9.4 ± 6.0	6.9 ± 3.6	12.7 ± 8.0	10.2 ± 5.2	< 0.001
Neutrophil count, × 10^9^/L	8.5 ± 9.2	5.7 ± 6.4	12.6 ± 11.9	8.6 ± 5.1	< 0.001
Lymphocyte count, × 10^9^/L	0.9 ± 0.6	1.0 ± 0.5	0.7 ± 0.8	0.9 ± 0.6	0.002
NLR (neutrophil/lymphocyte ratio)	13.1 ± 13.5	7.8 ± 9.3	21.3 ± 16.0	13.9 ± 13.0	< 0.001
Monocytes, count, × 10^9^/L	0.5 ± 0.4	0.5 ± 0.6	0.5 ± 0.4	0.5 ± 0.3	0.947
Platelet count, × 10^9^/L	214 ± 112	225 ± 97	180 ± 123	223 ± 118	0.001
C-reactive protein (mg/L)	78.7 ± 83.6	47.0 ± 51.4	116.1 ± 94.2	92.6 ± 93.8	< 0.001
Procalcitonin (ng/ml)	1.9 ± 8.8	0.7 ± 4.7	2.8 ± 10.5	2.7 ± 10.9	0.078
ALT (U/L)	47.1 ± 95.2	31.6 ± 30.2	80.8 ± 179.1	44.3 ± 40.9	< 0.001
AST (U/L)	60.2 ± 227.0	31.2 ± 25.0	110.7 ± 429.4	60.9 ± 138.3	0.019
Total bilirubin (μmol/L)	14.7 ± 11.5	11.0 ± 5.7	18.1 ± 13.2	16.8 ± 14.1	< 0.001
Direct bilirubin (μmol/L)	8.1 ± 7.5	5.0 ± 5.1	9.9 ± 9.2	10.6 ± 7.3	< 0.001
Albumin (g/L)	32.0 ± 5.6	32.7 ± 4.6	30.1 ± 7.0	32.4 ± 5.4	< 0.001
d-dimer (μg/mL)	5.9 ± 11.9	3.1 ± 5.3	13.2 ± 20.5	4.5 ± 7.0	0.276
Glucose (mmol/L)	8.7 ± 4.7	7.1 ± 3.3	10.3 ± 6.8	9.5 ± 3.9	< 0.001
Serum creatine (Scr) (μmol/L)	128.3 ± 190.7	124.5 ± 197.5	119.2 ± 165.2	140.2 ± 199.9	0.636
SOFA score on day 1	4.2 ± 3.1	2.2 ± 2.2	6.0 ± 3.0	5.5 ± 2.7	< 0.001
Continuous renal replacement therapy (CRRT), no. (%)	44 (9.4)	6 (2.9)	30 (26.5)	8 (5.3)	< 0.001
Extracorporeal membrane oxygenation (ECMO), no. (%)	10 (3.1)	0 (0.0)	10 (8.8)	0 (0.0)	< 0.001
Length of hospital stay (days)	20.4 ± 13.2	27.3 ± 14.7	17.9 ± 12.3	16.1 ± 9.6	< 0.001
Mortality, no. (%)	179 (38.2)	13 (6.4)	104 (92.0)	62 (40.8)	< 0.001

The mean age in the IV group was 71, which was significantly higher than the other two groups (67 in both the NIV group and NV group, *P* = 0.03). There were no significant differences in comorbidities on admission except chronic obstructive pulmonary disease (COPD). Interestingly, there were even more cases of COPD in the NIV group (31, 20.7%) than in the IV group (8, 7.1%). This could be explained that physicians tend to avoid intubation in chronic lung disease patients due to concern of barotrauma and higher DNR/DNI ratio in those patients. From laboratory results, significantly higher white blood cell count, lower lymphocyte count and platelet count, and higher CRP, AST, ALT, and total bilirubin are presented in the IV group than the other two groups on admission. SOFA scores in the IV and NIV groups were also significantly higher than the NV group. There were no significant differences in Scr among these groups on admission though. We can tell from the data that the patients in the IV group were older with a higher rate of hyperinflammation status on admission compared to the other two groups. These factors may lead to the rapid progress of respiratory failure and fatal outcome eventually [1].

Researchers worldwide also start to realize that IV may not improve the mortality in COVID-19 patients [2, 3]. We have to admit that some of the COVID-19 patients who developed progressive worsening respiratory distress were refractory to NIV. Intubation is inevitable in those cases. However, sometimes physicians can be rushed to intubation. It has been reported that intubation can be successfully avoided by HFNO [4, 5].

As we all know, IV can cause a lot of complications including hypotension, ventilator-related infection, volume imbalance, and sedation-related delirium. The decision of intubation mostly based on clinical judgments and varies from case to case. There is a notion that NIV causes wide-spread dispersion of aerosol thus increases the risk of transmission to healthcare workers. This could be one of the reasons that encourages physicians to choose IV over NIV among clinical decisions [5].

In conclusion, from our data in Wuhan, COVID-19 patients who were invasively ventilated exhibited pessimistic outcomes. This suggests that early intubation may not help patients but instead, make things head towards the wrong direction. We should try to avoid IV and utilize NIV at the early stage of respiratory failure until IV is inevitable [6]. It is time for physicians to rethink the threshold of intubation in COVID-19 management.

## Data Availability

The datasets used and/or analyzed during the current study are available from the corresponding author on reasonable request.

## Abbreviations

Covid-19: Coronavirus disease 2019
NV: No ventilation (nasal cannula oxygen)
NIV: Noninvasive ventilation (BiPAP, CPAP, or high-flow nasal oxygen)
IV: Invasive ventilation
HFNO: High-flow nasal oxygen
